# Catheter ablation of symptomatic idiopathic ventricular arrhythmias: A five-year single-centre experience

**DOI:** 10.1007/s12471-018-1085-5

**Published:** 2018-01-30

**Authors:** A. W. G. J. Oomen, L. R. C. Dekker, A. Meijer

**Affiliations:** Catharina Hospital, Eindhoven, The Netherlands

**Keywords:** Catheter, Ablation, Idiopathic, Premature, Ventricular, Tachycardia

## Abstract

**Aims:**

This study was designed to gain insight into the patient characteristics, results and possible complications of ablation procedures for symptomatic idiopathic premature ventricular complexes (PVC) and idiopathic ventricular tachycardia (VT).

**Methods:**

Data were collected from all patients who underwent radiofrequency catheter ablation for symptomatic PVCs and idiopathic VT in the Catharina Hospital between 1 January 2011 and 31 December 2015. The procedural endpoint was elimination or non-inducibility of the clinical arrhythmia. Successful sustained ablation was defined as the persistent elimination of at least 80% of the PVCs or the absence of VTs at follow-up. In case of suspected PVC-induced cardiomyopathy, the systolic left ventricular function was reassessed 3 months post procedure.

**Results:**

Our cohort consisted of 131 patients who underwent one or more ablation procedures; 99 because of symptomatic premature ventricular complexes, 32 because of idiopathic VT. In total 147 procedures were performed. The procedural ablation success rate was 89%. Successful sustained ablation rate was 82%. Eighteen (13.2%) patients had suspected PVC-induced cardiomyopathy. In 15 of them (83%), successful sustained ablation was achieved and the left ventricular ejection fraction improved from a mean of 39% (±8.8) to 55.4% (±8.1). Most arrhythmias originated from the right ventricular outflow tract (60%) or aortic cusps (13%). Complications included three tamponades.

**Conclusion:**

Catheter ablation therapy for idiopathic ventricular arrhythmias is very effective with a sustained success rate of 82%. In patients with PVC-induced cardiomyopathy, it leads to improvement of systolic left ventricular function. However, risk for complications is not negligible, even in experienced hands.

## What’s new?

- Catheter ablation for symptomatic premature ventricular beats and idiopathic ventricular tachycardia is very effective.
- In case of PVC-induced cardiomyopathy, the systolic LV function will improve after a successful catheter ablation.
- The complication rate of catheter ablation for the above-mentioned indications is, however, not negligible.
- Doctors should be aware of this treatment option in case of frequent PVCs or idiopathic VT, especially in patients with LV dysfunction.

## Introduction

Premature ventricular complexes (PVCs) and ventricular tachycardia (VT) occurring in the absence of structural heart disease are referred to as idiopathic ventricular arrhythmias. Frequent PVCs occur in 1–4% of the general population [1]. Usually these PVCs do not cause symptoms and patients without structural heart disease have a good prognosis [2]. However, some patients can be highly symptomatic [3, 4]. Furthermore, in the last decade, it has been increasingly recognised that frequent PVCs can lead to reduced systolic left ventricular (LV) function (PVC-induced cardiomyopathy) [4]. Idiopathic VT accounts for approximately 10% of all VT diagnoses [5]. Idiopathic ventricular arrhythmias occur in specific locations of the heart and the causative mechanisms can be triggered activity, re-entry or automaticity [6].

Medication is often used as the first-line option to suppress these idiopathic ventricular arrhythmias. This therapy is, however, often limited by lack of efficacy or not tolerated because of side effects [7].

Radiofrequency ablation of PVCs and idiopathic VT has evolved as a safe and effective treatment for symptomatic idiopathic PVCs/VT or PVC-induced cardiomyopathy. According to the 2015 European Society of Cardiology guidelines, catheter ablation is recommended in patients with symptomatic right ventricular outflow tract (RVOT) PVC/idiopathic VT after failed or not preferred antiarrhythmic medical therapy or if RVOT PVCs contribute to reduced LV ejection fraction (LVEF) (Class I, level of evidence B). In case of symptomatic PVCs/VT originating from the LVOT, aortic cusp or epicardium, catheter ablation should be considered after failed antiarrhythmic medical therapy (Class IIa, level of evidence B) [8].

In this study, we present the patient characteristics, results and complications of catheter ablation procedures for symptomatic idiopathic PVCs and idiopathic VT in a single high-volume centre.

## Methods

### Study population

This is a single-centre, observational study among consecutive patients who underwent a catheter ablation procedure because of symptomatic idiopathic PVCs or VT between 1 January 2011 and 31 December 2015. Patients were highly symptomatic and/or had presumed PVC-induced cardiomyopathy. Patients were not included if they were scheduled for an ablation but did not actually undergo this procedure. Patients were older than 18 years, able to read and sign informed consent. All patients underwent screening for structural heart disease prior to the ablation procedure. Patients with ischaemic heart disease, significant valvular disease, genetic or infiltrative cardiomyopathy were excluded from this database. The volume and function of the right ventricle were examined and ECGs were scrutinised to exclude any possible patients with arrhythmogenic right ventricular cardiomyopathy (ARVC). Significant coronary artery disease had to be ruled out by cardiac angiography or stress testing in all patients.

### LV function and PVC burden

Patients underwent a routine pre-procedural work-up by determining LV function and PVC burden. LV function was assessed by echocardiography using the Simpson formula or by MRI. When both techniques were used, we selected MRI ejection fraction. An LVEF below 51% was considered abnormal.

Also, Holter monitoring was performed at baseline to assess the PVC burden. This was done 3 months pre-procedure. PVC burden was defined as the number of PVCs as percentage of the total beats per 24 h.

### Ablation

All antiarrhythmic drugs were withdrawn at least 5 half-lives prior to the procedure except for amiodarone. All studies were performed by one of two experienced operators. PVCs or VTs were mapped using activation mapping if they occurred frequently or pace mapping if they were not frequent. If few or no PVCs were observed at baseline, programmed ventricular stimulation was performed with intravenous administration of isoproterenol to induce PVCs or VT. Mapping and ablation was guided by the CARTO electro-anatomic mapping system (Biosense Webster, DiamondBar, CA, USA) using a 3.5 mm irrigated-tip ablation catheter. From April 2012 on, contact-force ablation catheters were used. For left-sided procedures, systemic heparinisation was used to maintain an activated clotting time of 300–350 s. After ablation, patients were monitored for at least 30 min to ensure successful ablation. Procedural ablation success (PAS) was defined as elimination or non-inducibility of the clinical arrhythmia. One focus was treated in each procedure. After an effective ablation, all antiarrhythmic drugs were discontinued.

### Follow-up

Patients were routinely seen in the outpatient clinic 3 months after the procedure. Holter monitoring was repeated then in the majority of patients. Successful sustained ablation (SSA) was defined as the persistent elimination of at least 80% of the PVC burden or the absence of VTs. In patients in whom Holter recording was not available post ablation, absence of complaints was considered successful sustained ablation.

In case of LV dysfunction, assessment of LV function was also repeated after 3 months. PVC-induced cardiomyopathy was defined as a systolic LVEF of below 51% which improved after successful ablation.

### Statistical analysis

Continuous variables are presented as the mean ± standard deviation. PVC burdens are presented as the median and interquartile range due to their skewed distribution. Categorical variables are presented as total number and percentages. To compare two variables a paired t‑test or Wilcoxon signed-rank test was used. Proportions were compared using the Fisher’s exact test. A *p* value of <0.05 was considered statistically significant. All analyses were conducted using SPSS 22.0 software (IBM corporation, Armonk, NY).

## Results

In total 131 consecutive patients (mean age: 51 years) were included. Most patients were female (64%). All patients were symptomatic, had LV dysfunction or both. Ninety-nine patients (76%) had frequent PVCs, 32 patients (24%) presented with idiopathic VT. They underwent in total 147 ablation procedures. In one ablation procedure, the epicardium was targeted while all other procedures were endocardial ablations.

In 30 patients (23%), the ablation procedure was the first-line treatment. Drug treatments before the ablation procedure are shown in Tab. 1. Eight patients (6%) had already undergone a catheter ablation procedure for idiopathic VT or PVCs before 2011. These procedures were not recorded in this database.

**Table 1:** Baseline characteristics

Patients	131
Age (years)	51 (±10.4)
Male	47 (35.9)
Presenting with PVC/VT	99 (75.6)/32 (24.4)
Median PVC burden (%)	15.5 (9–30)
Previous PVC ablation	8 (6.1)
*Failed medical therapy*	
– Beta-blockers	60 (45.8)
– Calcium channel blockers	17 (13.0)
– Flecainide	9 (6.9)
– Combination of the above	17 (13.0)
– Amiodarone	1 (0.8)
*LV dysfunction*	18 (13.7)
– Mean LVEF in patients with LV dysfunction	39 (±8.8)
– Median PVC burden in patients with LV dysfunction	25.5 (12–33)

Values are mean ± SD, median (interquartile range) or *n* (%)

*PVC* premature ventricular complex, *VT* ventricular tachycardia, *LV* left ventricular, *LVEF* left ventricular ejection fraction

Procedural ablation success was achieved in 131 of the 147 ablation procedures (89%) (Tab. 2). In 10 procedures (7%) procedural ablation success was not accomplished, in 6 procedures (4%) this was unclear, mainly because of lack of PVCs at the start of the procedure.

**Table 2:** Results

*Total number of procedures*	147
– 1 procedure	118 (80.3)
– 2 procedures	11 (7.5)
– 3 procedures	1 (0.7)
– 4 procedures	1 (0.7)
Procedural ablation success	131 (89)
Unclear procedural ablation success	10 (15)
Successful sustained ablation	108 (82)
Successful sustained ablation in patients with LV dysfunction	15 (83)
Improvement of LVEF after successful sustained ablation in case of LV dysfunction	15 (100)
Mean LVEF post ablation in case of pre-existing LV dysfunction	55.4 (±8.1)
Median PVC burden after 1 or more ablation procedures	0.14 (0.01–1.1)
Median PVC burden after any procedural ablation success	0.1 (0.01–0.8)
Median PVC burden after successful sustained ablation	0.05 (0.01–0.3)
*Complications*	5 (3.8)
– Cardiac tamponade	3 (2.0)
– Abdominal haematoma	1 (0.7)
– Third-degree AV block	1 (0.7)
Mortality	0

Values are mean ± SD, median (interquartile range) or *n* (%)

*AV* atrioventricular, *PVC* premature ventricular complex, *VT* ventricular tachycardia, *LV* left ventricular, *LVEF* left ventricular ejection fraction

A single procedure was performed in 118 patients. In 11 patients, a second procedure was needed while there were 2 patients in whom a third and a fourth procedure were performed, respectively. The mean follow-up was 10.5 months (±7.3). Successful sustained ablation was achieved in 108 patients (82%).

**Fig. 1:**
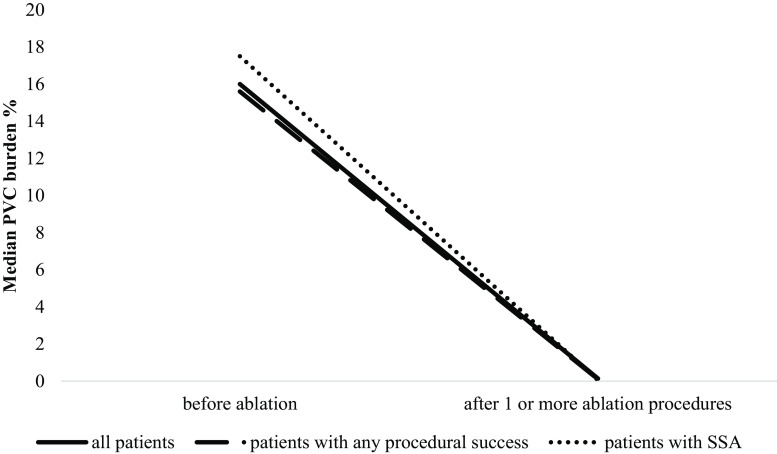
Median PVC burden before and after 1 or more ablation procedures

Holter registration both pre-and post-ablation was available in 90 of 99 patients with PVCs. The pre-procedural median PVC burden per 24 h in these patients was 15.5% (interquartile range 9–30). In patients with systolic LV dysfunction, the PVC burden was 25.5% (interquartile range 12–33). The median PVC burden after one or more ablation procedures was 0.14% (interquartile range 0.01–1.1). This decrease in PVC burden is significant (*p* = 0.001). The median burden at follow-up in patients with procedures with any procedural ablation success or successful sustained ablation was 0.1% (interquartile range 0.01–0.8) and 0.05% (interquartile range 0.01–0.3), respectively (Fig. 1).

In 13 patients, one or more redo procedures (in total 16) were performed. In 8 patients (9 redo procedures), the same region was targeted as during the previous procedure. In 4 of those patients, a lack of PVCs was reported during the first catheter ablation. In 3 patients (3 redo procedures), the site of origin was found to be located in the aortic cusp while during a previous procedure the RVOT was targeted. One patient underwent 3 redo procedures with eventually a successful endo-epicardial ablation. PVCs that differed from the previously ablated PVCs were found in 1 patient (1 redo procedure).

**Fig. 2:**
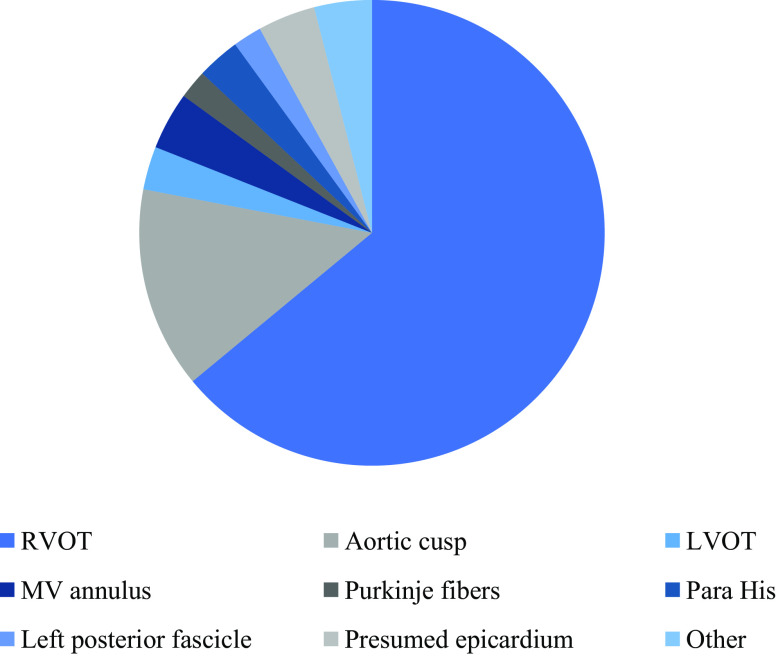
Distribution of sites of origin

The vast majority of PVCs and VT originated from the RVOT (88 procedures, 60%) with significantly less arrhythmias originating from the aortic cusps (19 procedures, 13%) and other parts of the heart such as the LV outflow tract, Purkinje fibres, epicardium or mitral valve annulus (Fig. 2).

**Fig. 3:**
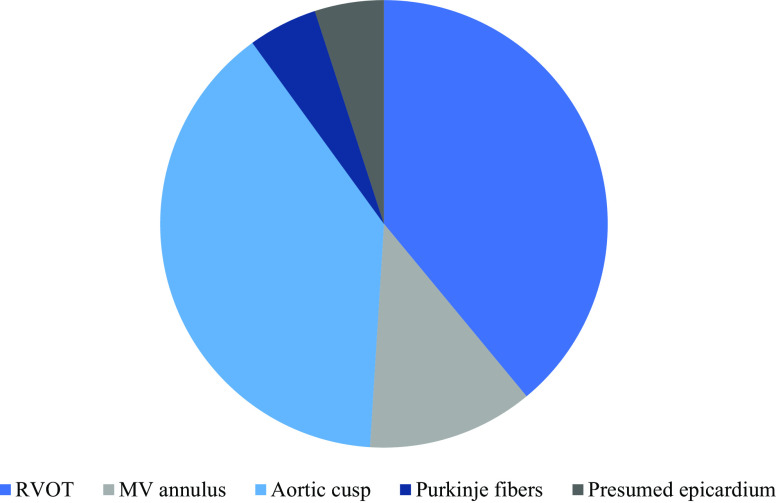
Distribution of sites of origin in case of left ventricular dysfunction

In patients with LV dysfunction, RVOT (*n* = 7, 39%) and aortic cusps (*n* = 7, 39%) constituted the most prevalent sites of origin (Fig. 3). This difference in distribution is not significant (*p* = 0.317).

There was no significant difference in successful sustained ablation with respect to the origin of the PVCs/idiopathic VT (*p* = 0.451). Contact-force ablation catheters were used in the majority of procedures (88%) with no statistical difference (*p* = 0.322) in the procedural outcome between the use of contact-force catheters and non-contact force catheters.

**Fig. 4:**
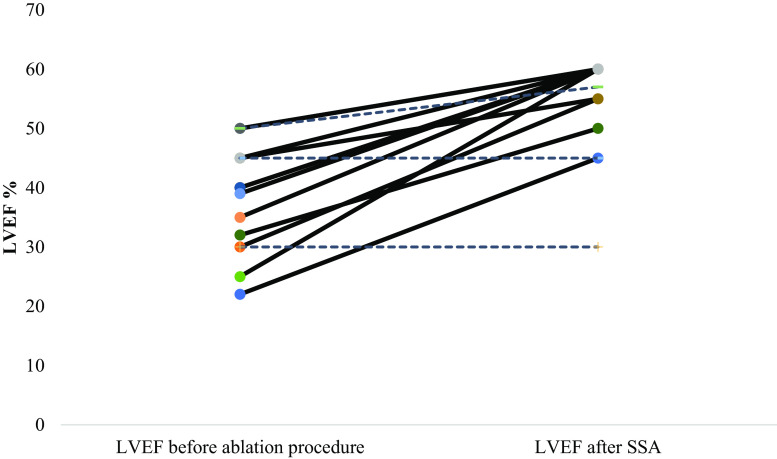
LVEF before and after one or more ablation procedures for PVCs in case of left ventricular dysfunction. Full lines show the LVEF for patients with sustained successful ablation (SSA), dashed lines show the LVEF for patients without SSA

Eighteen patients had LV dysfunction. The mean systolic LV function in those patients was 39% (±8.8). In 15 patients (83%) successful sustained ablation was achieved and in all those patients the LV function recovered. The systolic LV function in patients with pre-existent impaired LV function improved significantly to a mean of 55.4% (±8.1) (*p* = 0.001) after one or more ablation procedures (Fig. 4). In the patients without successful sustained ablation, the LV function did not change.

Of note is that the mean heart rate in the patients with LV dysfunction before (69 beats/min, ±7) and after ablation (70 beats/min, ±7) was not significantly different.

Complications occurred in 5 patients (3.8%): 3 tamponades of which 2 were drained percutaneously and 1 surgically, 1 with abdominal bleeding which was managed conservatively and 1 third-degree atrioventricular (AV) block requiring implantation of a permanent pacemaker. There were no fatal complications. Both the abdominal bleeding and the third-degree AV block were caused by using a non-contact force ablation catheter.

Two of the tamponades occurred while ablating a focus in the RVOT using a contact force catheter (one because of a steam pop). The other tamponade occurred using a non-contact force catheter ablating a focus at the mitral annulus.

## Discussion

The present study shows a high success rate for catheter ablation for symptomatic idiopathic ventricular arrhythmias with a procedural success rate of 89% and a sustained successful ablation rate of 82%. The percentage of successful sustained ablations in patients with LV dysfunction was 83% in our series. Importantly in all patients with LV dysfunction and successful sustained ablation, the LV function improved. The risk of complications is low though not negligible, even in experienced hands.

Our results are in accordance with earlier reports in the literature. Over the last years several studies on ablation for symptomatic idiopathic PVCs/VT have been published with success rates of over 80% and low complication rates [4, 7, 9, 10]. Results of PVC ablation in patients with LV dysfunction are also good. A meta-analysis showed long-term success rates of up to 80% in the majority of included studies and a relatively low risk of complications (no more than 8%) [11].

What this study adds to the aforementioned reports is that it includes a sizable real-life cohort of patients covering a long period of 5 years. Most other studies are smaller and focused solely on RVOT arrhythmias. Studies that report the results of catheter ablation of idiopathic PVCs/VTs from other origins are sparse and the majority included fewer patients. This study included all-comers and was not restricted to a certain site of origin of the arrhythmia. Therefore, it gives relevant insights into real-life practice of catheter ablation of idiopathic ventricular arrhythmias.

Successful ablation was associated with improvements in LV function and LV dimensions, both in our study and in the literature [4, 10, 12, 13]. The improvement in LV function presumably has prognostic consequences. This improvement is not only true for patients with PVC-induced cardiomyopathy but also for patients with ‘PVC worsened’ cardiomyopathy [14, 15]. In other words; the improvement of LV function after successful ablation of frequent PVCs may be independent of the presence of structural heart disease.

The underlying mechanism of PVC-induced cardiomyopathy is uncertain. Ventricular dyssynchrony and irregularity are potential causes. One of the initial hypotheses was that PVC-induced cardiomyopathy was a tachycardia-induced cardiomyopathy. However, in this study, the mean heart frequency in the patients with improved LV function was not significantly different before and after ablation. The heart rate during the pre-procedure Holter registrations may have been influenced by the use of antiarrhythmic drugs while all Holter registrations following ablation procedures were performed in patients off any antiarrhythmic drugs.

It is also not entirely clear yet how to identify those patients with LV dysfunction who will benefit from PVC ablation. Patients with wider PVC complexes, higher PVC burdens (from 10%) and a longer duration of symptoms might benefit more. Some studies suggest that PVCs from the right ventricle carry more risk to develop LV dysfunction but these findings could not be confirmed in larger studies [11, 16, 17].

The ventricular outflow tracts are the most common origins of idiopathic PVC/VT [5, 9]. Also in our series, the majority of our patients presented with outflow tract arrhythmias. Remarkable is the difference in distribution of sites of origin between patients with normal versus diminished LV function. Due to the spread and the relatively low numbers, this is not significantly different but it is something worth investigating further.

In our study, complications occurred in 5 patients, with tamponade in 3 of them. This relatively high percentage of tamponade may be due to the learning curve and the fragility of the RVOT. There was no significant difference in the risk of tamponades between contact force versus non-contact force ablation catheters. It is important to note that there were no fatal complications and that the percentage of complications is in line with other studies. For example, Valk et al. reported 4% haemodynamically important pericardial effusions after 82 ablations of idiopathic PVCs and VTs in the outflow tracts using non-contact force ablation catheters [18].

Steam pops occur infrequently in ventricular ablation. In a series of over 4,000 ablation lesions, steam pops occurred in 62 lesions (1.5%). Larger falls in impedance and higher maximum energy are associated with pops [19].

The mean follow-up in this study was 10.5 months which may be relatively short. We cannot exclude recurrences occurring after the follow-up period. There are no guidelines about how long the follow-up should be. However, given that this treatment is solely intended to reduce symptoms and is a curative treatment in patients without structural heart disease, we feel there is no need for longer follow-up in those patients.

### Limitations

Several limitations are worth noting. This was a single-centre study with one of two experienced operators performing the ablation procedures. Outcomes may therefore differ in other centres with lower volumes. Due to the study design patients were not included, and therefore not reported, in this study if no PVCs or VTs were seen or inducible during the procedure. There is no uniformly accepted definition of successful sustained ablation. Therefore, results may differ in other studies using another definition of this endpoint. Defining success by at least 80% reduction of PVCs after the ablation procedure was introduced previously by Penela et al. [14]. As this procedure is primarily aimed at reducing symptoms, we added the absence of symptoms to this definition.

Another limitation is that Holter follow-up data were not available for all patients. Nonetheless, we think that also the subjective results in patients without Holter follow-up are important because the ablation procedure is primarily aimed at reducing symptoms.

In most procedures, a contact-force ablation catheter was used. Although we did not find any statistical differences in outcome with the different types of ablation catheters, it is not unimaginable that there would be a difference if more non-contact force catheters had been used.

We tried our best to exclude patients with structural heart disease. However, the diagnosis of ARVC only relies partially on imaging and ECG criteria. Therefore, although unlikely, we cannot entirely exclude the possibility that some patients with ARVC may have been included in this database.

Finally, in 2 patients with LV dysfunction, the ablation was not successful. In those patients, the LV function did not change after the procedure. We cannot exclude dilated cardiomyopathy that was not related to frequent PVCs in those patients.

Although this is a retrospective study with its inherent limitations, we studied consecutive patients representing a real-world cohort of patients who are candidates for ablation according the current guidelines. We therefore think that our findings are valuable for current everyday practice.

## Conclusion

In this study we demonstrated that catheter ablation is an effective and relatively safe treatment for patients with symptomatic frequent PVCs or idiopathic VT. Furthermore, patients with LV dysfunction due to the arrhythmia can be cured by successful catheter ablation. This presumably has prognostic consequences for these patients. It is therefore important that doctors are aware of this treatment option in case of frequent PVCs or idiopathic VT, especially in patients with LV dysfunction.

## References

[1] Kostis JB, McCrone K, Moreyra AE (1981). Premature ventricular complexes in the absence of identifiable heart disease. Circulation.

[2] Kennedy HL, Whitlock JA, Sprague MK (1985). Long-term follow-up of asymptomatic healthy subjects with frequent and complex ventricular ectopy. N Engl J Med.

[3] Van Huls van Taxis CF, Piers SR, de Riva SM (2015). Fatigue as presenting symptom and a high burden of premature ventricular contractions are independently associated with increased ventricular wall stress in patients with normal left ventricular function. Circ Arrhythm Electrophysiol.

[4] Bogun F, Crawford T, Reich S (2007). Radiofrequency ablation of frequent, idiopathic premature ventricular complexes: comparison with a control group without intervention. Heart Rhythm.

[5] Calvo N, Jongbloed M, Zeppenfeld K (2013). Radiofrequency catheter ablation of idiopathic right ventricular outflow tract arrhythmias. Indian Pacing Electrophysiol J.

[6] Lerman BB, Stein KM, Markowitz SM (1997). Mechanisms of idiopathic ventricular tachycardia. J Cardiovasc Electrophysiol.

[7] Ling Z, Liu Z, Su L (2014). Radiofrequency ablation versus antiarrhythmic medication for treatment of ventricular premature beats from the right ventricular outflow tract: prospective randomized study. Circ Arrhythm Electrophysiol.

[8] Priori SG, Blomström-Lundqvist C, Mazzanti A (2015). ESC Guidelines for the management of patients with ventricular arrhythmias and the prevention of sudden cardiac death. Eur Heart J.

[9] Adams JC, Srivathsan K, Shen WK (2012). Advances in management of premature ventricular contractions. J Interv Card Electrophysiol.

[10] Lamba JL, Redfearn DP, Michael KA, Simpson CS, Abdollah H, Baranchuk A (2014). Radiofrequency catheter ablation for the treatment of idiopathic premature ventricular contractions originating from the right ventricular outflow tract: a systematic review and meta-analysis. Pacing Clin Electrophysiol.

[11] Zang M, Zhang T, Mao J, Zhou S, He B (2014). Beneficial effects of catheter ablation of frequent premature ventricular complexes on left ventricular function. Heart.

[12] Yokokawa M, Good E, Crawford T (2013). Recovery from left ventricular dysfunction after ablation of frequent premature ventricular complexes. Heart Rhythm.

[13] Wijnmaalen AP, Delgado V, Schalij MJ (2010). Beneficial effects of catheter ablation on left ventricular and right ventricular function in patients with frequent premature ventricular contractions and preserved ejection fraction. Heart.

[14] Penela D, Van Huls Van Taxis C, Aguinaga L (2013). Neurohormonal, structural, and functional recovery pattern after premature ventricular complex ablation is independent of structural heart disease status in patients with depressed left ventricular ejection fraction: a prospective multicenter study. J Am Coll Cardiol.

[15] El Kadri M, Yokokawa M, Labounty T (2015). Effect of ablation of frequent premature ventricular complexes on left ventricular function in patients with nonischemic cardiomyopathy. Heart Rhythm.

[16] Del Carpio Munoz F, Syed FF, Noheria A (2011). Characteristics of premature ventricular complexes as correlates of reduced left ventricular systolic function: study of the burden, duration, coupling interval, morphology and site of origin of PVCs. J Cardiovasc Electrophysiol.

[17] Baman TS, Lange DC, Ilg KJ (2010). Relationship between burden of premature ventricular complexes and left ventricular function. Heart Rhythm.

[18] Valk SD, de Groot NM, Szili-Torok T (2012). Clinical characteristics and acute results of catheter ablation for outflow tract ventricular tachycardia or premature beats. J Interv Card Electrophysiol.

[19] Seiler J, Roberts-Thomson KC, Raymond JM (2008). Steam pops during irrigated radiofrequency ablation: feasibility of impedance monitoring for prevention. Heart Rhythm.

